# Long non‐coding RNA H19 mediates osteogenic differentiation of bone marrow mesenchymal stem cells through the miR‐29b‐3p/DKK1 axis

**DOI:** 10.1111/jcmm.18287

**Published:** 2024-04-29

**Authors:** Sen Qin, Da Liu

**Affiliations:** ^1^ Department of Orthopedics Shengjing Hospital of China Medical University Shenyang Liaoning China

**Keywords:** bone marrow mesenchymal stem cells, lncRNA H19, osteogenic differentiation, osteoporosis, spinal cord injury

## Abstract

Single immobilization theory cannot fully account for the extensive bone loss observed after spinal cord injury (SCI). Bone marrow mesenchymal stem cells (BMSCs) are crucial in bone homeostasis because they possess self‐renewal capabilities and various types of differentiation potential. This study aimed to explore the molecular mechanism of long non‐coding RNA H19 in osteoporosis after SCI and provide new research directions for existing prevention strategies. We used small interfering RNA to knockdown H19 expression and regulated miR‐29b‐2p expression using miR‐29b‐3p mimetics and inhibitors. Western blotting, real‐time fluorescence quantitative PCR, Alizarin red staining, alkaline phosphatase staining and double‐luciferase reporter gene assays were used to assess gene expression, osteogenic ability and binding sites. lncRNA H19 was upregulated in BMSCs from the osteoporosis group, whereas miR‐29b‐3p was downregulated. We identified the binding sites between miR‐29b‐3p and lncRNAs H19 and DKK1. H19 knockdown promoted BMSCs' osteogenic differentiation, whereas miR‐29b‐3p inhibition attenuated this effect. We discovered potential binding sites for miR‐29b‐3p in lncRNAs H19 and DKK1. Our findings suggest that long non‐coding RNA H19 mediates BMSCs' osteogenic differentiation in osteoporosis after SCI through the miR‐29b‐3p/DKK1 axis and by directly inhibiting the β‐catenin signalling pathway.

## 1 INTRODUCTION

Various motor, sensory and sphincter abnormalities; aberrant muscle tone; and autonomic dysfunction may arise from spinal cord injury (SCI), which can be caused by direct or indirect external sources.^1^ The risk of a series of paralysis‐related complications is significantly increased in patients with SCI, with severe and rapid bone density loss being the most pronounced manifestation.^2^, ^3^ The main reason for this phenomenon is the lack of mechanical stimulation, which leads to a decrease in the ability of osteoblasts to form and an increase in the bone resorption ability of osteoclasts.^4^, ^5^ A single explanation cannot explain such substantial bone loss, deterioration of bone structure or impairment of bone biomechanical characteristics because the clinical signs of secondary osteoporosis caused by SCI are more specific.^6^ Changes in the body's nervous system and physiological dysfunction contribute to the loss of bone density after SCI.^7^, ^8^ Some classic signalling pathways have been shown to be involved in the loss of bone density, but the molecular and cellular mechanisms driving this type of osteoporosis have not been confirmed.^9^, ^10^ Therefore, a comprehensive investigation into the molecular pathways involved in the development of osteoporosis following SCI will help identify new therapeutic targets for the prevention and rehabilitation of osteoporosis.

Bone marrow mesenchymal stem cells (BMSCs) are a type of stem cell with self‐renewal and multi‐directional differentiation ability.^11^ It is also a pioneer in cell treatment and tissue engineering because of its homing ability and precise differentiation at the damage site.^12^ One cause of osteoporosis is the disruption of the balance between osteogenic and adipogenic differentiation of BMSCs.^13^ Therefore, because BMSCs are considered precursor cells of osteoblasts, they are used as seed cells for the treatment of osteoporosis.^14^ The molecular process of the osteogenic differentiation of BMSCs has not been fully recognized. Therefore, further research on the osteogenic process of BMSCs will help develop new methods for preventing and treating osteoporosis after SCI.

Long non‐coding RNAs (lncRNAs) are a type of RNA that usually contain 200 or more nucleotides.^14^, ^15^ Numerous studies have demonstrated the critical regulatory roles of lncRNAs in various diseases and biological processes.^16^, ^17^ Recent studies have highlighted the significance of lncRNAs in BMSCs' osteogenic differentiation.^18^, ^19^ MicroRNAs (miRNAs) are a class of short non‐coding RNAs that regulate gene expression by pairing with the 3′ untranslated region of a target mRNA.^20^ One of the mechanisms by which lncRNAs compete for sponging miRNAs as endogenous RNAs is sequence complementarity, which reduces the functional availability of targeted miRNAs. This lncRNA‐miRNA interaction forms a regulatory network that controls downstream target gene expression.^21^

Many studies have indicated that H19 is involved in the occurrence and development of osteoporosis.^22^, ^23^ However, there have been no reports of this particular type of osteoporosis after SCI. Therefore, it is necessary to gain a deep understanding of the molecular mechanisms underlying osteoporosis to better prevent its occurrence. Consistent with a previous study, we found that H19 was significantly upregulated in BMSCs in a rat model of osteoporosis after SCI.^24^ In addition, bioinformatics analysis indicated that H19 competitively inhibits miR‐29b‐3p by targeting DKK1. Low H19 expression increases the level of miR‐29b‐3p and decreases the expression of DKK1. Therefore, we speculated that the H19‐miR‐29b‐3p‐DKK1 axis plays a crucial role in osteoporosis after SCI by regulating the differentiation of BMSCs.

This study aimed to explore the molecular mechanism of H19 in osteoporosis after SCI and provide new research directions for existing prevention strategies.

## 2 MATERIALS AND METHODS

### 2.1 Osteoporosis model establishment and isolation and identification of BMSCs

Specific pathogen‐free Sprague–Dawley male, 6‐week‐old rats (*n* = 16) (Huafu Kang, Beijing, China) were randomly divided into two groups: SHAM (*n* = 8) and SCI (*n* = 8). The rats in the SCI group underwent total spinal cord transection at the lumbar 10–12 segments after 1 week of acclimation in an SPF‐level holding chamber. In contrast, the SHAM group only had the corresponding segment's vertebrae removed. Throughout the 12‐week study period, each rat was housed individually in a separate cage under a 12/12‐h light/dark cycle. They were provided ad libitum access to de‐rodentized chow and drinking water.

After 12 weeks, all rats with SCI developed osteoporosis and were euthanized, and their femurs and tibias were extracted. BMSCs were isolated using the bone marrow cavity flushing technique.^25^ Extracted BMSCs were cultured in Dulbecco's modified Eagle medium/nutrient mixture F‐12 (DMEM/F12, Thermo Fisher, USA) supplemented with 15% foetal bovine serum. Additionally, 1% penicillin–streptomycin solution (Pricella, Wuhan, China) was added to the culture medium. The cells were incubated in a 5% CO_2_, 37°C cell incubator. The cells were cultured until the third generation for further experiments.

Cells cultured to the third‐generation were analysed by flow cytometry using antigen–antibody‐specific binding (BD Biosciences) with antibodies against CD29 (BD Biosciences), CD45 (BD Biosciences) and CD90 (BD Biosciences).

### 2.2 Induction of osteogenic differentiation of BMSCs

BMSCs were seeded at a density of 1 × 10^5^ cells/well in a 6‐well plate and induce osteogenic differentiation when the cell growth density reached 70%–80%. The osteoblastic differentiation medium was prepared by supplementing Dulbecco's modified Eagle medium/nutrient mixture F‐12 (DMEM/F12, Thermo Fisher, USA) with 10% foetal bovine serum, 1% penicillin–streptomycin solution (Prisella, Wuhan, China), 200uM ascorbic acid (Sigma, USA), 10 mM sodium glycerophosphate (Sigma, USA) and 100 nM dexamethasone (Sigma, USA). The cells were incubated in a cell incubator at 37°C with 5% CO_2_. The culture medium was replaced every 3 days for a total of 14 days. When observed under a microscope, the cells exhibited various morphologies, including single cells, spindle‐shaped cells, fibrous‐like cells, network‐like cells, vortex‐like cells, fish‐like cells and radiating‐shaped cells, indicating the presence of BMSCs.

### 2.3 RNA extraction and qRT‐PCR assay

Total RNA was extracted from BMSCs using TRIzol reagent (Invitrogen, USA). The purity and concentration of RNA were determined using UV spectroscopy. The TransScript One‐Step gDNA Removal and cDNA Synthesis SuperMix kit (TransGen Biotech, Beijing, China) was used to reverse‐transcribe 2 μg of the total RNA into cDNA. An ABI Prism 7500 Rapid Real‐Time PCR system (Applied Biosystems, StepOnePlus, USA) and TransStart Green qPCR SuperMix kit (TransGen Biotech, Beijing, China) were used to measure the expression levels of H19, miR‐29b‐3p, RUNX2, OCN and OPN. U6 small nuclear RNA (U6 snRNA) and β‐actin were used as internal references for miRNA and mRNA, respectively. The relative expression level was determined using the 2^−ΔΔCt^ method. All primers used in the quantitative real‐time PCR (qRT‐PCR) analysis were synthesized by Sangon Biotech (Shanghai, China). The primer sequences used for qRT‐PCR are listed in Table 1.

**TABLE 1:** The primer sequences of gennes in this experiment.

Target genes	Forward primer	Reverse primer
LncRNA H19	GGCAGGTGAGTCTCCTTCTT	TCCTGCCTTTCTATGTGCCA
miR‐29b‐3p	CGCGTAGCACCATTTGAAATC	AGTGCAGGGTCCGAGGTATT
Runx2	GTTCCCAGGCATTTCATCCC	AAGGTGGCTGGATAGTGCAT
OCN	GACCCTCTCTCTGCTCACTC	GGGCTCCAAGTCCATTGTTG
OPN	GCCGAGGTGATAGCTTGGCTTA	TTGATAGCCTCATCGGACTCCTG
Dkk1	CTCTGTCTGCCTCCGATCAT	CTCCTGTGCTTGGTGCATAC
β‐actin	CACCATGTACCCAGGCATTG	CCTGCTTGCTGATCCACATC
U6	CGGGTTTGTTTTGCATTTCT	AGTCCCAGCATGAACAGCTT

Abbreviations: Dkk1, Dickkopf‐related protein 1; OCN, osteocalcin; OPN, osteopontin; Runx2, runt‐related transcription factor 2.

### 2.4 Western blot analysis

Western blotting was performed as previously described.^23^ RIPA protein extraction reagent (Beyotime, Beijing, China) was used to lyse BMSCs, followed by treatment with protease (Beyotime, Beijing, China) and phosphatase inhibitors (Beyotime, Beijing, China). The concentrations of isolated proteins were determined using a BCA Protein Assay Kit (Beyotime, Beijing, China). Thirty micrograms of total protein was electrophoretically separated by 10% sodium dodecyl sulphate‐polyacrylamide gel electrophoresis (SDS‐PAGE, Solarbio, Beijing, China) and then transferred to polyvinylidene difluoride membranes (PVDF, Solarbio, Beijing, China). The membranes were incubated with 5% bovine serum albumin (BSA, Solarbio, Beijing, China) for 2 h. Primary antibodies against β‐catenin, GSK‐3β, DKK1, RUNX2, OSX, OPN, OCN and TCF‐1 were applied to the membranes and incubated overnight at 4°C. Details of the manufacturers, dilutions and product numbers of the antibodies are shown in Table S1. The membranes were then incubated with horseradish peroxidase (HRP)‐conjugated goat anti‐rabbit secondary antibody (Abbkine, Wuhan, China) for 2 h at 37°C. Subsequently, the bands were visualized using an Enhanced Chemiluminescence Kit (Epizyme Biomedical, Shanghai, China), and the results were evaluated using Quantity One Imaging Software (Bio‐Rad, CA, USA).

### 2.5 Cell transfection

The si‐lncRNA H19, rno miR‐29b‐3p mimetics and their negative controls (ncs) were synthesized by Biomics Biotech (Nantong, China), and the miR‐29b‐3p inhibitor and inhibitor‐nc were synthesized by Genesyntech (Nantong, China). BMSCs were transfected using the Lipofectamine 3000 reagent (Invitrogen) following the manufacturer's instructions. The sequences are listed in Table S2.

### 2.6 Alkaline phosphatase (ALP) staining and activity

After inducing osteogenic differentiation for 7 days, the cells were collected and fixed with 4% paraformaldehyde (Solarbio, Beijing, China) for 30 min. The cells were washed thrice for 1 min each. The cells were then incubated with BCIP/NBT Color Reagent Kit (Coolaber, Beijing, China) for 20 min. Staining was observed under an inverted microscope (Leica DMIRB). The black and grey granular positions were regarded as calcium deposits, indicating the intensity of ALP staining.^26^ ALP activity was determined using an ALP colorimetric analysis kit (Elab Science, Wuhan, China) and normalized to total protein content. ALP staining and quantitative experiments were repeated 3 times for each section; A total of 39 images were captured throughout the entire experiment.

### 2.7 Alizarin red S (ARS) staining and quantitative analysis

After inducing osteogenic differentiation for 14 days, the cells were collected and fixed with 4% paraformaldehyde (Solarbio, Beijing, China) for 30 min and washed thrice for 1 min each. Incubate the cells with 2% pH 4.2 Alizarin Red staining solution (Solarbio, Beijing, China) at room temperature in dark for 20 min. Staining was observed under an inverted microscope (Leica DMIRB). To quantify the degree of mineralization of BMSCs, the stain was solubilized with cetylpyridinium chloride (Solarbio, Beijing, China) and quantified using a spectrophotometer at 570 nm.^26^ ARS staining and quantitative experiments were repeated 3 times for each section; a total of 39 images were captured throughout the entire experiment.

### 2.8 Immunofluorescence assay

Cells were cultured on spherical coverslips in 12‐well plates at a density of 2 × 10^4^ cells/well. To permeabilize the cells, 0.5% Triton X‐100 (Solarbio, Beijing, China) was applied, followed by fixation with 4% paraformaldehyde (Solarbio, Beijing, China) at room temperature for 30 min. Subsequently, the cells were incubated with anti‐RUNX2 in a moist box at 4°C overnight after being blocked with 5% BSA for 30 min. After treatment with a Cy3‐labelled goat anti‐rabbit fluorescent IgG secondary antibody (Absin, Shanghai, China), the cells were stained with DAPI (Absin, Shanghai, China). Fluorescence microscopy (Eclipse NI; Nikon, Tokyo, Japan) was used to observe the staining.

### 2.9 Dual‐luciferase reporter gene assay

A targeted association between miR‐29b‐3p and 3′‐UTR of H19 or DKK1 was detected using dual‐luciferase reporter gene detection. The 3′‐UTR fragment of the WT H19 sequence or DKK1 mRNA was amplified and inserted into the pmiRGLO dual‐luciferase miRNA target expression vector (GenePharma, Shanghai, China), and pmiRGLO‐H19‐WT or pmiRGLO‐Dkk1‐WT was constructed. The GeneArt™ Site‐Directed Mutagenesis PLUS System (Thermo Fisher, USA) was adopted to mutate the putative binding site of the miR‐29b‐3p family in H19 or Dkk1 3′‐UTR. MUT H19 or Dkk1 3′‐UTR was inserted into the pmiRGLO carrier to form pmiRGLO‐H19‐MUT or pmiRGLO‐Dkk1‐MUT. HEK293T cells were purchased from the Shanghai Institute of Cell Biology of the Chinese Academy of Sciences (Shanghai, China). HEK293T cells were seeded in 96‐well plates at 60% confluence. miRNAs and dual‐luciferase vectors were co‐transfected with Liposome 3000 reagent (Invitrogen, USA). After 48 h, the cells were lysed, and luciferase activity was measured using the same method as the dual‐luciferase reporter detection system (GenePharma, Shanghai, China) according to the manufacturer's specifications.^25^

### 2.10 BMSC transplantation in rats with SCI

Rats with SCI were created by performing a complete spinal cord transection. Then, 18 rats were randomly divided into three groups: SHAM group, SCI group and SCI+lnc H19 (−) group, with six rats in each group. In the SCI+lnc H19 (−) group, rats were injected with 5 × 10^6^ lentivirus‐transfected BMSCs via the tail vein at 4, 6, 8 and 10 weeks after the operation.^25^ BMSCs were suspended in 1 mL PBS. In the nc group, BMSCs transfected with shRNA1‐NC were injected. The rats in the control group received PBS injections. After 12 weeks, the lower limbs were removed and stored in 4% paraformaldehyde (Solarbio, Beijing, China).

### 2.11 Statistical analysis

All experiments were independently conducted at least thrice, including one representative experiment. The data were analysed using Prism 8.0 software (GraphPad, USA) and expressed as means ± standard deviations (SD). Independent sample *t*‐tests were used to evaluate the statistical differences between the two groups, while one‐way ANOVA was employed to analyse multiple groups of data. Statistical significance was set at *p* < 0.05.

## 3 RESULTS

### 3.1 Characterization of osteoporotic BMSCs and H19 and miR‐29b‐3p dysregulation

**FIGURE 1:**
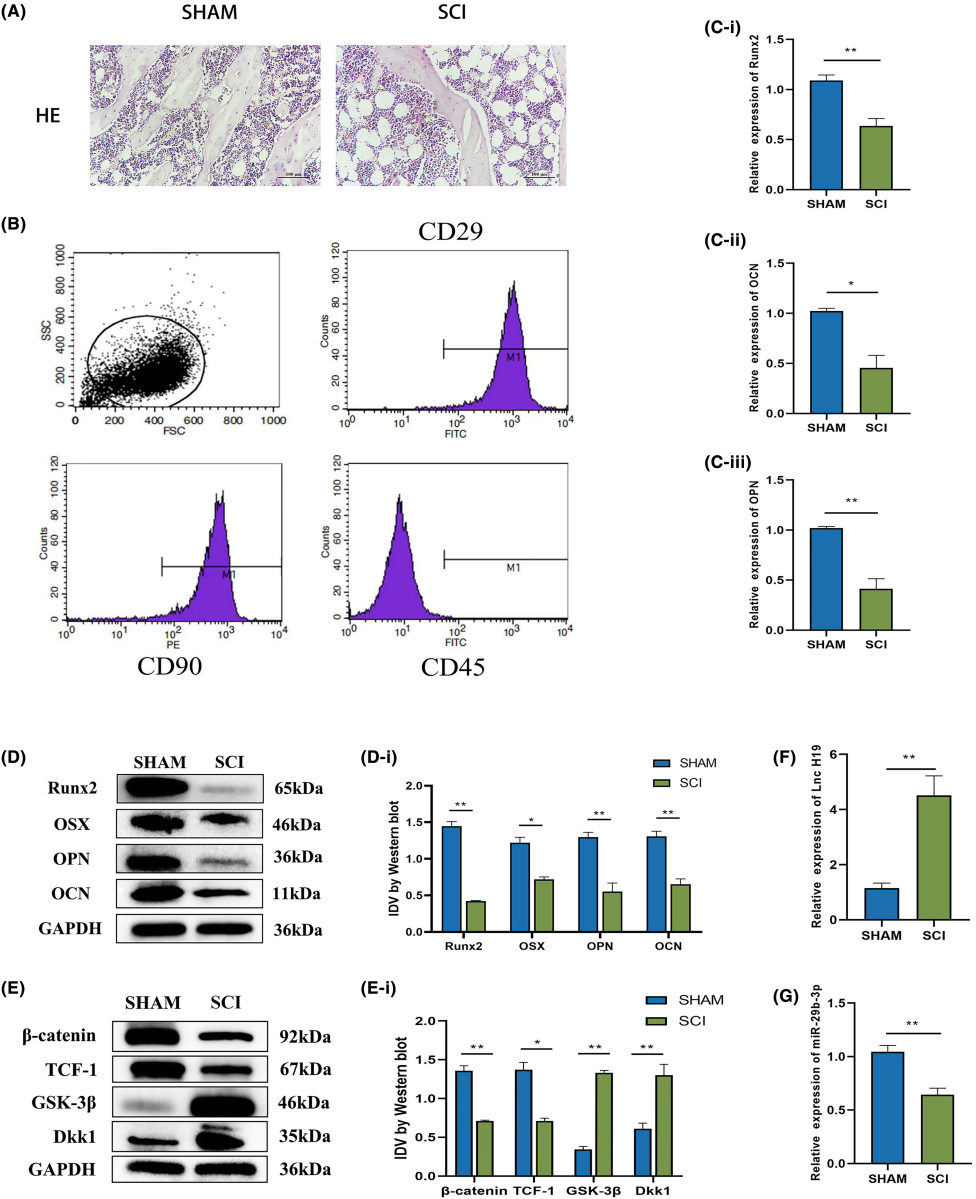
Expression of lncRNA H19 was upregulated in osteoporosis bone marrow mesenchymal stem cells, while the expression of miR‐29b‐3p was downregulated. (A) Representative images of HE staining from the SHAM and SCI rat groups showed that, when compared with SHAM, SCI rats had bone loss. (B) Extracted BMSCs were identified using flow cytometry. The positivity rate for CD29 was 98.41%, positivity rate for CD45 was 1.14%, and positivity rate for CD90 was 99.72%. (C) The expression levels of osteogenic differentiation indices (RUNX2, OPN and OCN) in BMSCs of SHAM and SCI rats were detected by qRT‐PCR. (D, E) Osteogenic differentiation index (RUNX2, OSX, OPN, OCN) and Wnt/β‐catenin channel indicators (β‐catenin, TCF‐1, GSK‐3β, DKK1) in the SHAM and SCI groups. (F, G) Expression levels of lncH19 and miR‐29b‐3p in BMSCs of the SHAM and SCI groups were detected by qRT‐PCR. Data are presented as the means ± SEM (A: *n* = 8; B–G: *n* = 3 per group). *p < 0.05, **p < 0.01, ***p < 0.001 VS SHAM group.

Recent studies have suggested that abnormal osteogenic differentiation of BMSCs contributes to the pathogenesis of osteoporosis. To investigate the pathological mechanisms underlying osteoporosis, we used a complete spinal cord transection model to induce osteoporosis. Histological analysis of the distal femur using haematoxylin and eosin (HE) staining revealed a significant reduction in bone trabeculae in the SCI group compared to that in the SHAM group (Figure 1A). Subsequently, we isolated bone marrow mesenchymal stem cells from SHAM and SCI rats and performed flow cytometry analysis to identify third‐generation cultured cells. Flow cytometry confirmed that the isolated cells exhibited characteristic markers of BMSCs, further validating their identity (Figure 1B). Compared with those in the SHAM group, the expression levels of recombinant runt‐related translation factor 2 (RUNX2), osteopontin (OPN), osteocalcin (OCN) and osterix (OSX) in the SCI group were significantly reduced (Figure 1C,D). In addition, the expression of β‐catenin and transcription factor 1 (TCF‐1) was downregulated in the SCI group, whereas the pathway inhibitors GSK‐3β and DKK1 exhibited opposite results (Figure 1E). Furthermore, our investigation revealed that the expression of H19 was upregulated, while miR‐29b‐3p expression was downregulated in osteoporotic BMSCs (Figure 1F,G). These findings strongly suggest that H19 and miR‐29b‐3p play crucial roles in the disruption of the osteogenic differentiation of osteoporotic BMSCs.

### 3.2 H19, miR‐29b‐3p and osteoblast gene expression during in vitro osteogenic differentiation of BMSCs

**FIGURE 2:**
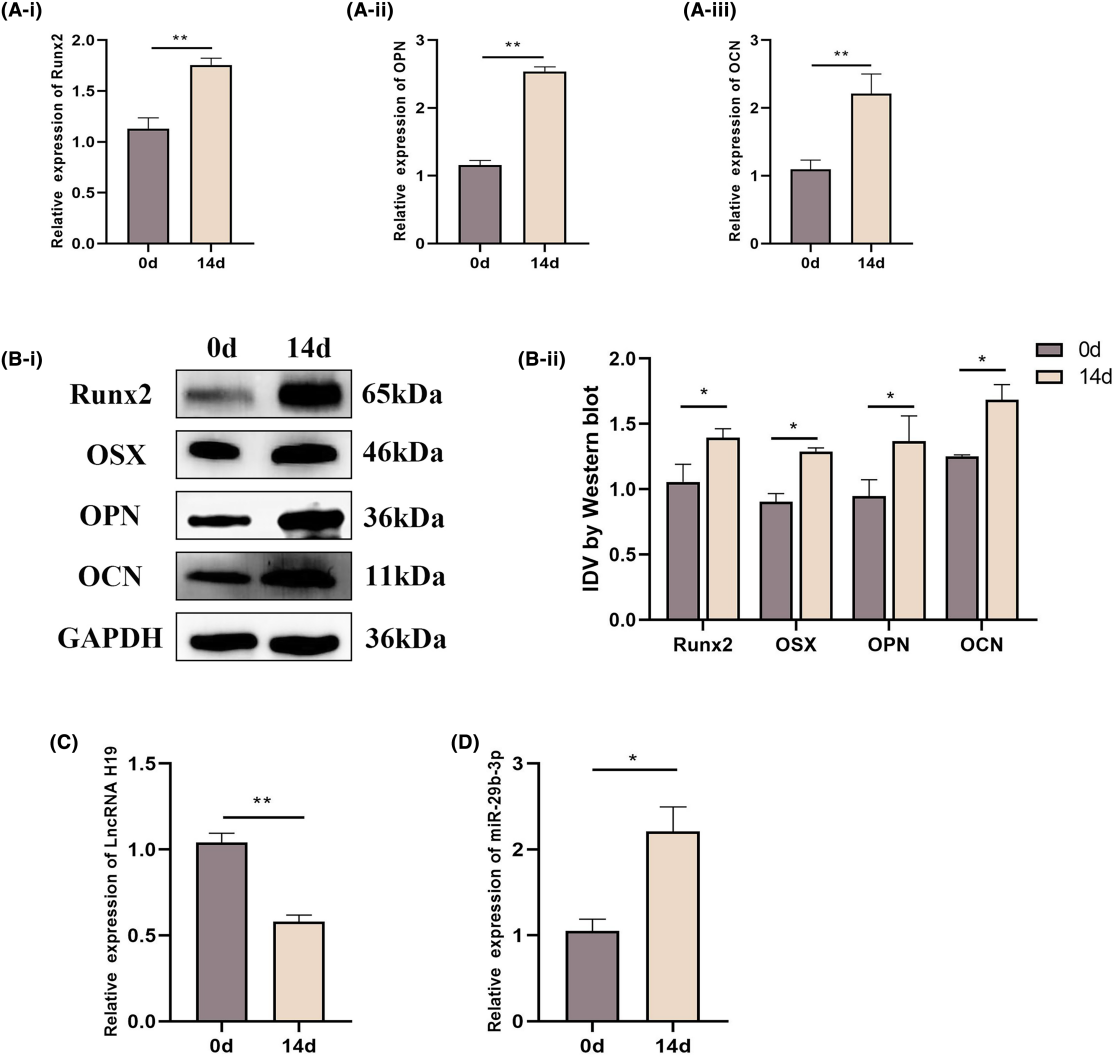
Expression levels of lncRNA H19, miR‐29b‐3p and osteoblast genes during in vitro osteoblastic differentiation. (A) After BMSCs were incubated in osteoblast induction medium for 0 and 14 days, mRNA expression levels of osteogenic differentiation indices (RUNX2, OCN and OPN) were detected by qRT‐PCR. (B) After BMSCs were incubated in osteoblast induction medium for 0 and 14 days, the protein expression levels of osteogenic differentiation indices (RUNX2, OPN and OCN) were detected by western blotting. (C, D) After BMSCs were incubated in osteoblastic induction medium for 0 and 14 days, the expression levels of lncRNA H19 and miR‐29b‐3p were detected by qRT‐PCR. Data are presented as the means ± SEM (*n* = 3 per group). *p < 0.05, **p < 0.01; VS 0‐days group.

We induced osteogenesis in BMSCs for 14 days and evaluated the mRNA expression of osteogenic genes to determine whether the levels of H19 and miR‐29b‐3p changed during this process. After 14 days of induction, the expression of these genes was significantly increased (Figure 2Ai–iii). Western blotting results showed that after 14 days of induction, the protein expression of these osteogenic genes significantly increased (Figure 2Bi,ii). We examined the expression of H19 and miR‐29b‐3p during osteogenic differentiation. According to the qRT‐PCR data, miR‐29b‐3p expression increased, whereas H19 expression decreased during osteogenic differentiation (Figure 2C,D).

### 3.3 H19 knockdown can effectively promote osteogenic differentiation of BMSCs

**FIGURE 3:**
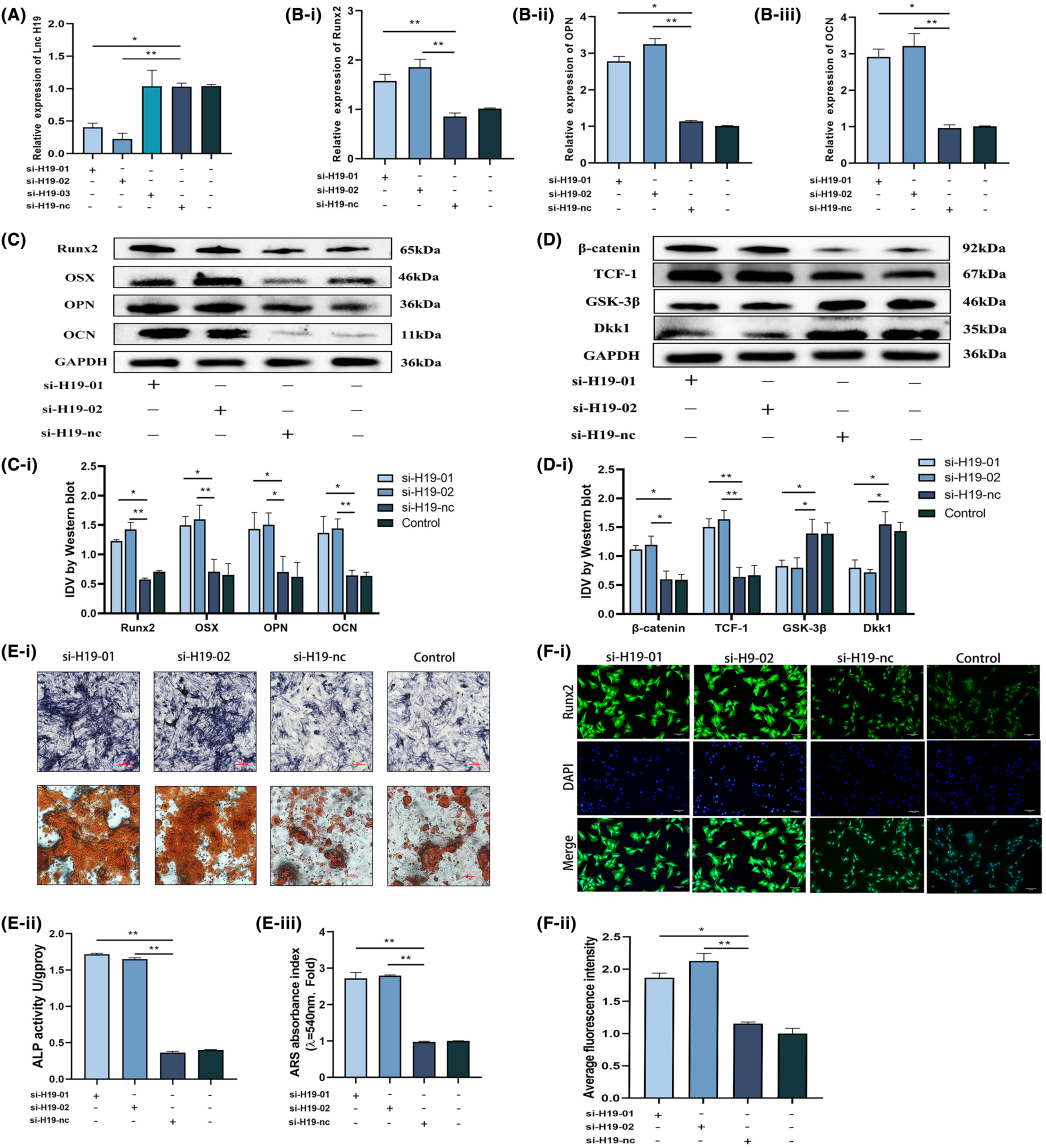
Knockdown of lncRNA H19 significantly improved the osteogenic differentiation ability of BMSCs. (A) The knockdown efficiency of siRNA H19 was verified by qRT‐PCR. (B) The effect of H19 knockdown on the osteogenic differentiation indices (RUNX2, OCN and OPN) of BMSC cultured in osteoblastic medium for 14 days was detected by qRT‐PCR. (C, D) The protein expression level of osteogenic differentiation index (RUNX2, OSX, OPN, OCN) and Wnt/β‐catenin pathway index (β‐catenin, TCF‐1, GSK‐3β, DKK1) of BMSC cultured in osteoblastic medium for 14 days after lncRNA H19 knockdown. (E) After the knockdown of lncRNA H19, BMSCs were cultured in osteoblastic medium for 7 days (image magnification: ×100) and then stained with alkaline phosphatase and alizarin red after 14 days (image magnification: ×200). (F) Immunofluorescence of the osteogenic index RUNX2 7 days after BMSC osteogenesis induction after lncRNA H19 knockdown (image magnification: ×100) Data are presented as the means ± SEM (*n* = 3 per group). *p < 0.05, **p < 0.01, ***p < 0.001; VS nc group.

We transfected BMSCs with a small interfering RNA (si‐H19) specifically designed to target H19 to determine how it affects the osteogenic development of BMSCs. qRT‐PCR was used to confirm the effectiveness of si‐H19's transfection in BMSCs (Figure 3A). According to the qRT‐PCR data, after inducing osteogenic differentiation in BMSCs for 14 days, H19 knockdown successfully increased the expression of the osteogenic marker mRNA (Figure 3Bi–iii). The outcomes of the western blotting experiment revealed that the H19 knockdown dramatically raised the expression levels of the osteogenic marker proteins, β‐catenin and TCF‐1 (Figure 3C,D). However, the expression of GSK‐3β and DKK1 was significantly downregulated (Figure 3C,D). The production of mineralized nodules in BMSCs 14 days after osteogenic differentiation was increased by transfection with si‐H19 to knockdown H19 expression, as shown in Figure 3E, in contrast to the negative control group (nc), which did not exhibit any discernible alterations (Figure 3E–I). After 7 days of osteogenic development, alkaline phosphatase (ALP) staining studies showed that BMSCs treated with si‐H19 presented more pronounced staining, indicating a greater degree of differentiation into mature osteoblasts (Figure 3E–I). Quantitative analysis of ARS staining combined with ALP activity confirmed that H19 knockdown significantly promoted the osteogenic differentiation of BMSCs (Figure 3Eii,iii). After 14 days of osteogenic development, immunofluorescence labelling and quantitative studies showed that si‐H19‐treated BMSCs had considerably higher levels of the osteogenic marker RUNX2 expression (Figure 3Fi,ii). These findings imply that the Wnt/β‐catenin signalling pathway mediates the effects of H19 knockdown by efficiently enhancing the differentiation of BMSCs into osteoblasts.

### 3.4 H19 targets miR‐29b‐3p, whereas miR‐29b‐3p can regulate osteogenic differentiation of BMSCs

**FIGURE 4:**
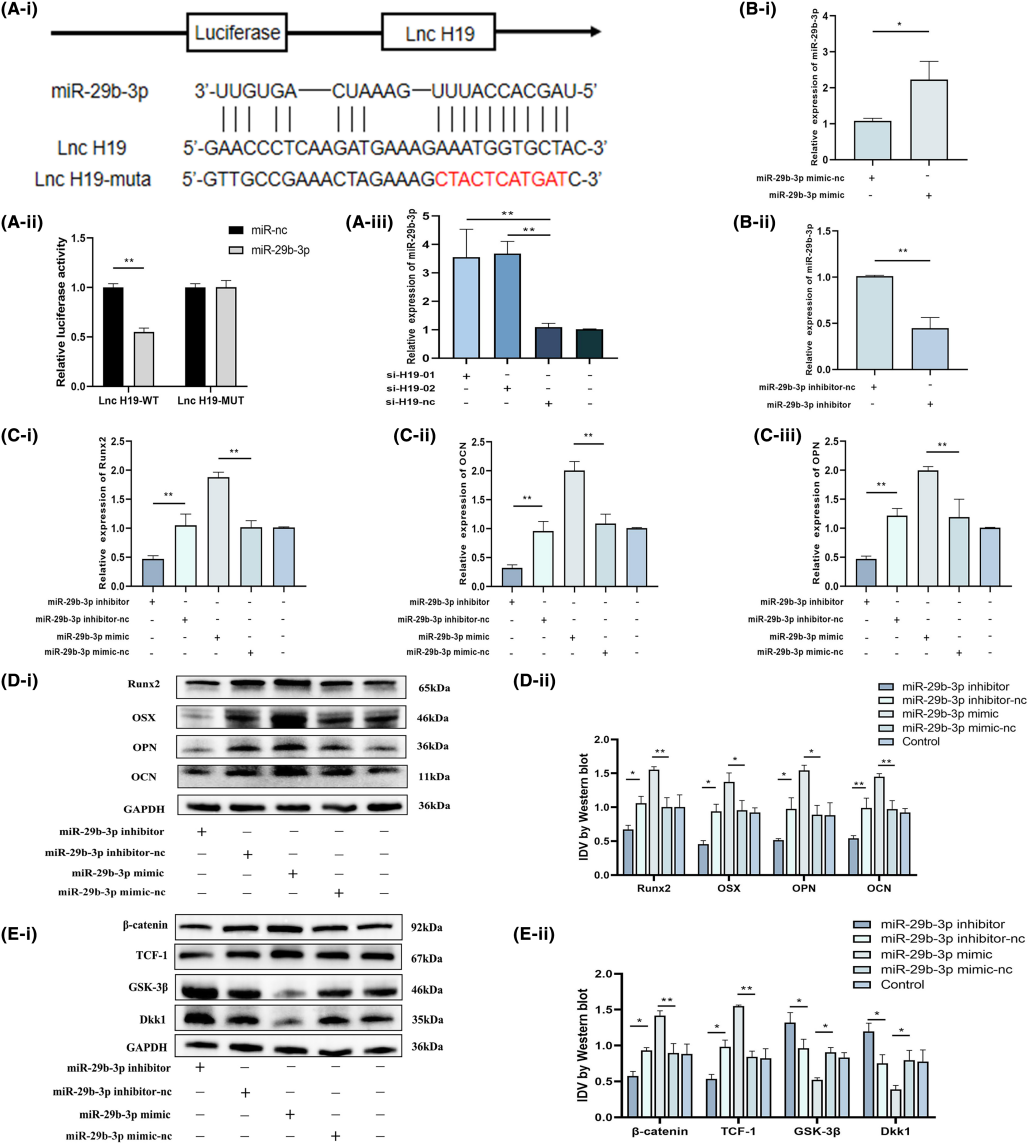
LncRNA H19 can target miR‐29b‐3p and participate in regulating the osteogenic differentiation of BMSCs. (A) Predicted binding sites of lncRNA H19 and miR‐29b‐3p were verified using double‐luciferase reporter gene analysis. After the expression of lncRNA H19 was altered, the relative expression of miR‐29b‐3p was detected. (B) Detected transfection efficiency when transfecting the miR‐29b‐3p mimic and inhibitor. (C) Effects of miR‐29b‐3p on osteogenic differentiation indices (RUNX2, OCN and OPN) of BMSC cultured in osteoblastic medium for 14 d at high and low expression levels were detected by qRT‐PCR. (D, E) The protein expression level of osteogenic differentiation index (RUNX2, OSX, OPN, OCN) and Wnt/β‐catenin pathway index (β‐catenin, TCF‐1, GSK‐3β, DKK1) of BMSC after 14 days of culture in an osteoblastic medium when miR‐29b‐3p is high‐ and low‐expressed. Data are presented as the means ± SEM (*n* = 3 per group). *p < 0.05, **p < 0.01; VS nc group.

Previous research has shown that H19 plays a critical role in controlling BMSCs' osteogenic differentiation. To further elucidate the underlying mechanisms, we investigated the potential regulatory pattern of H19, known as competitive endogenous RNA, which is commonly observed among various lncRNAs involved in gene expression regulation. Through predictive analysis, we identified a potential target interaction between H19 and miR‐29b‐3p (Figure 4A–I). Therefore, we speculated that miR‐29b‐3p may play a role in the osteogenic differentiation of BMSCs triggered by H19. A dual‐luciferase reporter assay was used to examine this process in detail. While the MUT vector showed no change in luciferase activity, transfection with the miR‐29b‐3p mimic dramatically decreased luciferase activity induced by H19‐WT (Figure 4A‐ii). Additionally, we observed that knocking down H19 contributes to the expression of miR‐29b‐3p when we altered the expression of H19 (Figure 4A‐iii). If miR‐29b‐3p mediates the regulatory effect of H19 on the osteogenic differentiation of BMSCs, then we speculated that its effect on the osteogenic differentiation of BMSCs may be opposite to that of H19. We conducted functional investigations utilizing an miR‐29b‐3p inhibitor and an miR‐29b‐3p mimic to reduce or increase the expression of miR‐29b‐3p in BMSCs to examine this (Figure 4Bi,ii).

**FIGURE 5:**
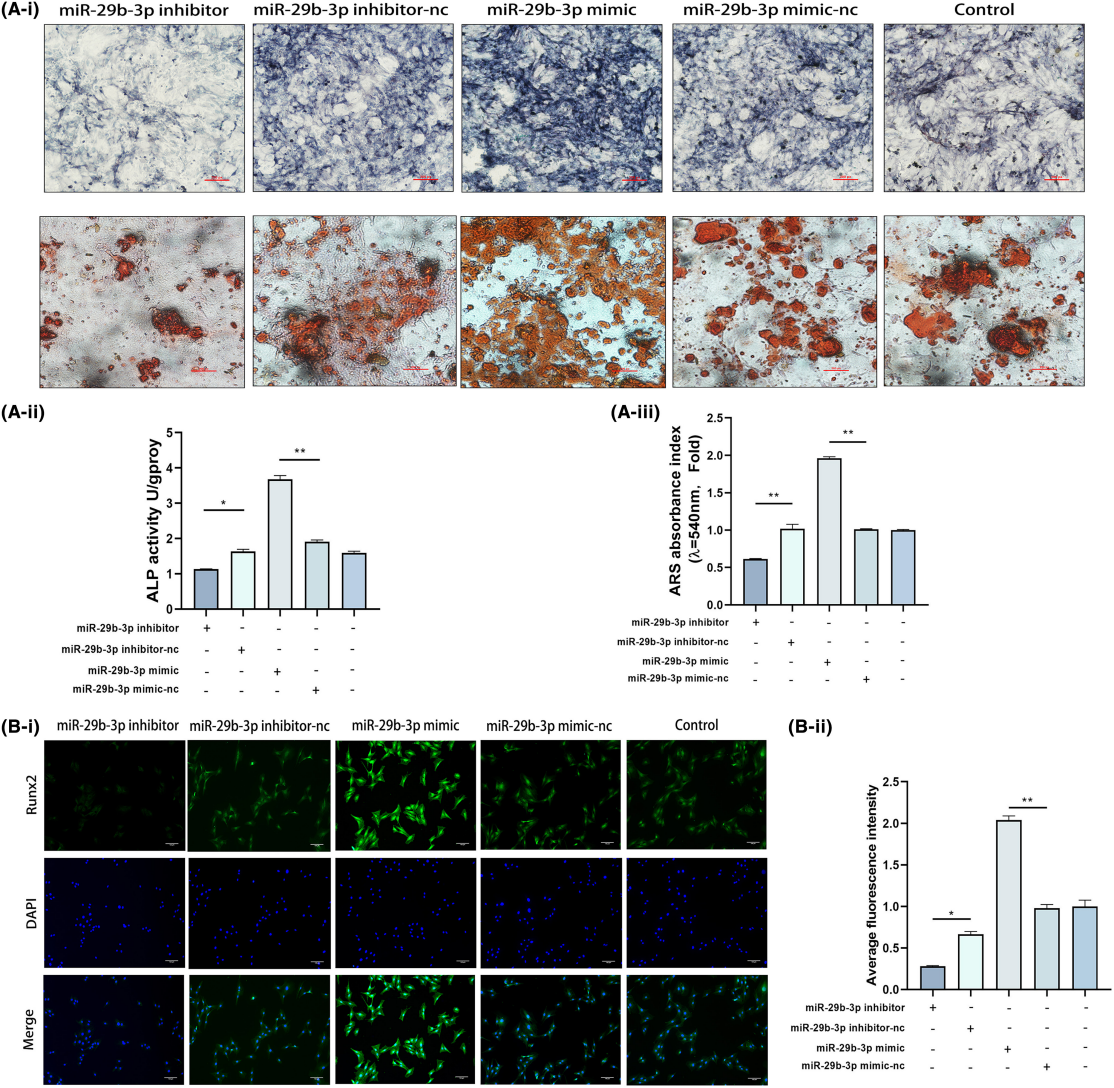
Low expression of H19 contributes to osteogenic differentiation of BMSCs. (A) miR‐29b‐3p was stained for alkaline phosphatase after 7 days of culture in osteogenic medium (image magnification: ×100) versus Alizarin Red staining after 14 days of culture (image magnification: ×200) at high and low expression of miR‐29b‐3p. (B) Immunofluorescence of the osteogenic index RUNX2 after 7 days of BMSC osteogenesis induction with high and low expression of miR‐29b‐3p (image magnification: ×100). Data are presented as the means ± SEM (*n* = 3 per group). *p < 0.05, **p < 0.01; VS nc group.

Following transfection with the miR‐29b‐3p inhibitor, the mRNA expressions of RUNX2, OCN and OPN were considerably reduced in BMSCs that had been grown in an osteogenic medium for 14 days. In contrast, the miR‐29b‐3p mimic had the opposite effect (Figure 4Ci–iii). Similarly, low expression of miR‐29b‐3p significantly inhibited the expression of β‐catenin and TCF‐1, while the expression of pathway inhibitor proteins (GSK‐3β, DKK1) was significantly increased (Figure 4D,E). However, the overexpression of miR‐29b‐3p produced the opposite result, with increased expression of β‐catenin and TCF‐1 and decreased expression of GSK‐3β and DKK1 (Figure 4D,E). After 7 days of osteogenic differentiation induction in transfected BMSCs, ALP activity was significantly increased in the miR‐29b‐3p overexpression group, while the opposite trend was observed in the miR‐29b‐3p low expression group (Figure 5A–I). ARS staining showed that knocking down miR‐29b‐3p successfully prevented the development of mineralized nodules, while the overexpression group of miR‐29b‐3p significantly increased the number of mineralized nodules (Figure 5A–I). In addition, the results of the ALP and ARS quantitative analyses were consistent with the staining results (Figure 5Aii,iii). The immunofluorescence results showed that the overexpression of miR‐29b‐3p significantly increased the expression of RUNX2, whereas the opposite was observed in the group with low miR‐29b‐3p (Figure 5Bi,ii).

### 3.5 Dkk1 is a candidate target gene of miR‐29b‐3p, which mediates the H19‐miR‐29b‐3p axis

**FIGURE 6:**
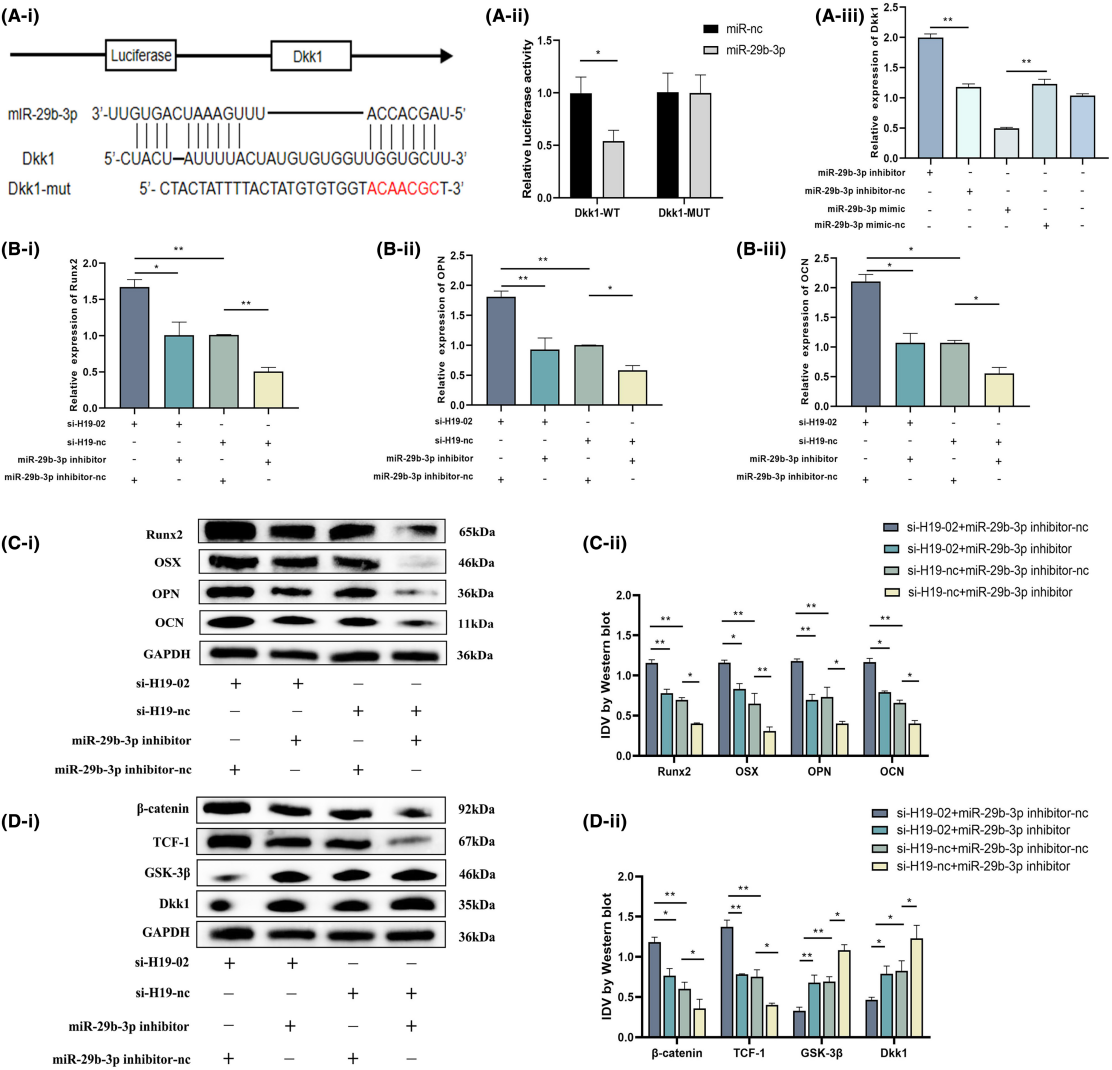
LncRNA H19 regulates the osteogenic differentiation of BMSCs by targeting miR‐29b‐3p, while Dkk1 is a candidate target gene of miR‐29b‐3p. (A) Predicted binding sites of miR‐29b‐3p and DKK1 were verified using double‐luciferase reporter gene detection. Relative mRNA expression of Dkk1 is regulated by miR‐29b‐3p. (B) Relative expression of osteogenic differentiation indicator mRNAs (RUNX2, OCN and OPN) in si‐H19 co‐transfected BMSC with the miR‐29b‐3p inhibitor after 14 days of culture in osteogenic medium. (C, D) Protein expression levels of osteogenic differentiation indicators (RUNX2, OSX, OPN, OCN) and Wnt/β‐catenin pathway indicators (β‐catenin, TCF‐1, GSK‐3β, DKK1) in si‐H19 co‐transfected BMSC with miR‐29b‐3p inhibitor after 14 days of culture in osteogenic medium. Data are presented as the means ± SEM (*n* = 3 per group). *p < 0.05, **p < 0.01, ***p < 0.001; VS nc group.

We evaluated the effect of miR‐29b‐3p on important BMSC osteogenic differentiation target genes to better study the mechanism by which the H19‐miR‐29b‐3p axis controls BMSC osteogenic differentiation. We identified Dkk1 as a candidate miR‐29b‐3p target based on previous studies and database predictions (https://mirdb.org/cgi‐bin/search.cgi). To verify this interaction, we created a luciferase reporter vector, as shown in Figure 6A–I. While no changes were observed in the MUT vector after transfection with the miR‐29b‐3p mimic, the luciferase activity caused by the Dkk1‐WT construct was significantly reduced (Figure 6A‐ii). We further altered the expression of miR‐29b‐3p and found that the expression of DKK1 was significantly reduced when miR‐29b‐3p was highly expressed, while the opposite was observed in the low expression group of miR‐29b‐3p (Figure 6A‐iii). In addition, we observed a substantial drop in DKK1 levels in the miR‐29b‐3p mimic group compared to those in the nc group after 14 days of osteogenic differentiation, whereas DKK1 levels were greater in the miR‐29b‐3p inhibitor group (Figure 4Ei,ii).

### 3.6 Downregulation of miR‐29b‐3p inhibits the positive effect of H19 downregulation on osteogenic differentiation of BMSCs

**FIGURE 7:**
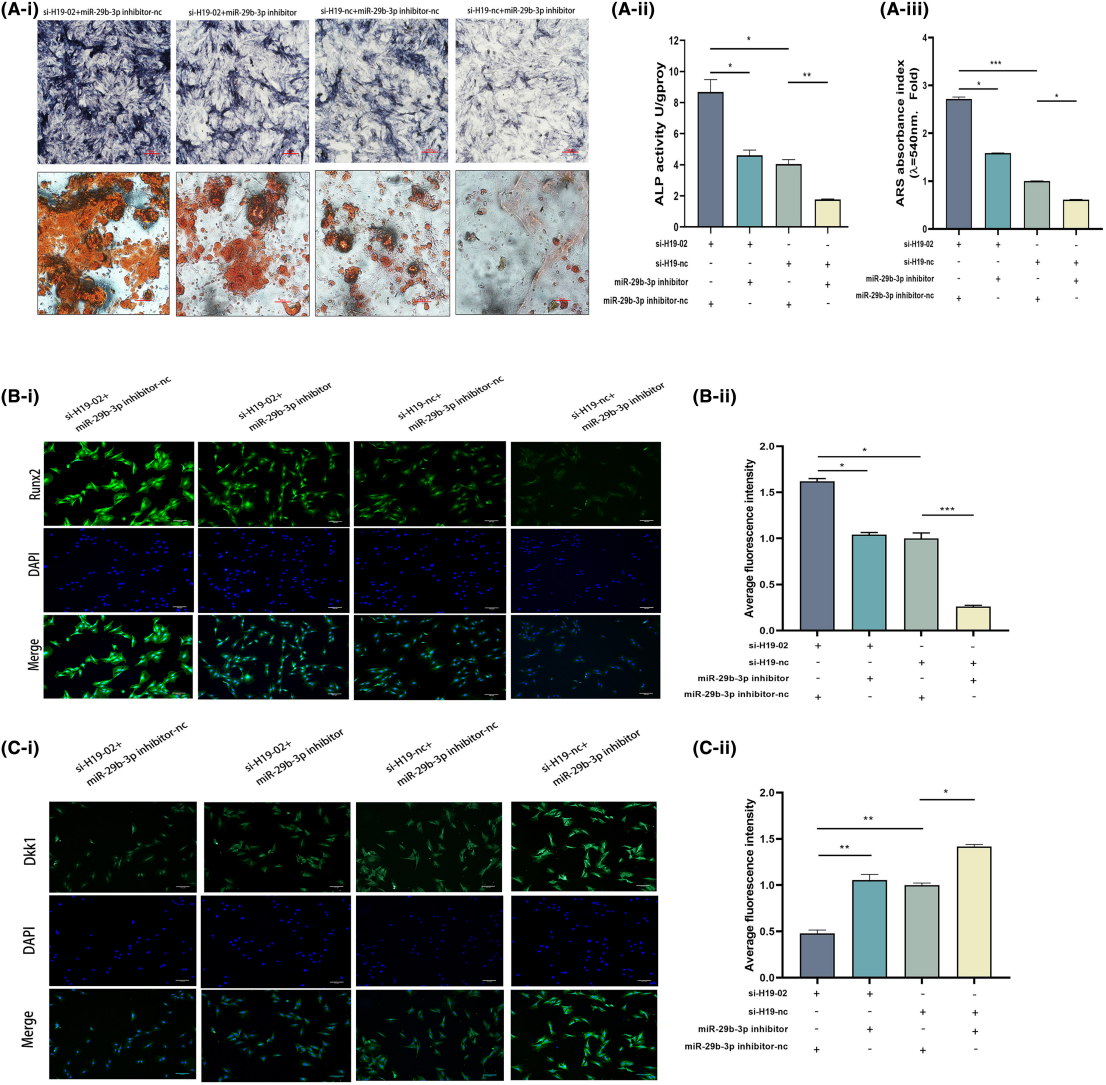
Low expression of miR‐29b‐3p can effectively reverse the effect of H19 low expression on promoting bone differentiation in BMSCs. (A) si‐H19 co‐transfected BMSC with miR‐29b‐3p inhibitor was stained for alkaline phosphatase after 7 days of culture in osteogenic medium (image magnification: ×100) versus alizarin red staining after 14 days of culture (image magnification: ×200). (B) Immunofluorescence of the osteogenic index RUNX2 in si‐H19 co‐transfected BMSC with miR‐29b‐3p inhibitor 7 days after osteogenic induction (image magnification: ×100). (C) Immunofluorescence of the target protein DKK1 in si‐H19 and miR‐29b‐3p inhibitor co‐transfected BMSC; after 7 days of osteogenesis induction (image magnification: ×100). Data are presented as the means ± SEM (*n* = 3 per group). *p < 0.05, **p < 0.01, ***p < 0.001; VS nc group.

We performed reparative studies to examine the regulatory function of the H19‐miR‐29b‐3p‐DKK1 axis in the osteogenic differentiation of BMSCs. The following groups of BMSCs were created after co‐transfection with si‐H19 and miR‐29b‐3p inhibitor: si‐H19‐02+ miR‐29b‐3p inhibitor‐nc group, si‐H19‐02+ miR‐29b‐3p inhibitor group, si‐H19‐nc+ miR‐29b‐3p inhibitor group and si‐H19‐nc+ miR‐29b‐3p inhibitor group. The si‐H19‐02+ miR‐29b‐3p inhibitor‐nc group showed the highest expression levels of osteogenic markers, including RUNX2, OPN and OCN, 14 days after osteogenic differentiation induction in the transfected BMSCs. However, the expression levels returned to normal in the group that included the miR‐29b‐3p inhibitor and si‐H19‐02 (Figure 6Bi,iii). Western blotting results showed that the relative protein expression of osteogenic indicators such as RUNX2, OSX, OPN, OCN, β‐catenin and TCF‐1 was highest in the si‐H19‐02+ miR‐29b‐3p inhibitor‐nc group but was restored in the si‐H19‐02+ miR‐29b‐3p inhibitor group (Figure 6C,D). However, the opposite results were observed for the pathway inhibitory proteins (GSK‐3β and DKK1) (Figure 6C,D). Transfected BMSCs were cultured in osteogenic media for 7 days. The si‐H19‐02+ miR‐29b‐3p inhibitor‐nc group showed more intense ALP staining than the si‐H19‐02+ miR‐29b‐3p inhibitor group (Figure 7A–I). The production of mineralized nodules was more prevalent in the si‐H19‐02+ miR‐29b‐3p inhibitor‐nc group according to the results of ARS staining but was recovered in the si‐H19‐02+ miR‐29b‐3p inhibitor group after 14 days of osteogenic induction in the transfected BMSCs (Figure 7A–I). Quantitative analyses of ALP and ARS revealed similar results (Figure 7Aii,iii). The conclusion drawn from the immunofluorescence analysis of RUNX2 was consistent with the above results (Figure 7Bi,ii). Owing to the ability of miR‐29b‐3p to sponge DKK1's mRNA to interfere with DKK1 expression, DKK1 expression was reduced in the miR‐29b‐3p high expression group. Similarly, H19 sponges miR‐29b‐3p; thus, co‐transfection effectively reversed the low expression of DKK1 caused by miR‐29b‐3p (Figure 7Ci,ii). Thus, the H19‐miR‐29b‐3p‐DKK1 axis is crucial for controlling the differentiation of BMSCs into osteoblasts.

### 3.7 BMSCs transfected with shRNA1‐H19 delay osteoporosis progression after SCI

**FIGURE 8:**
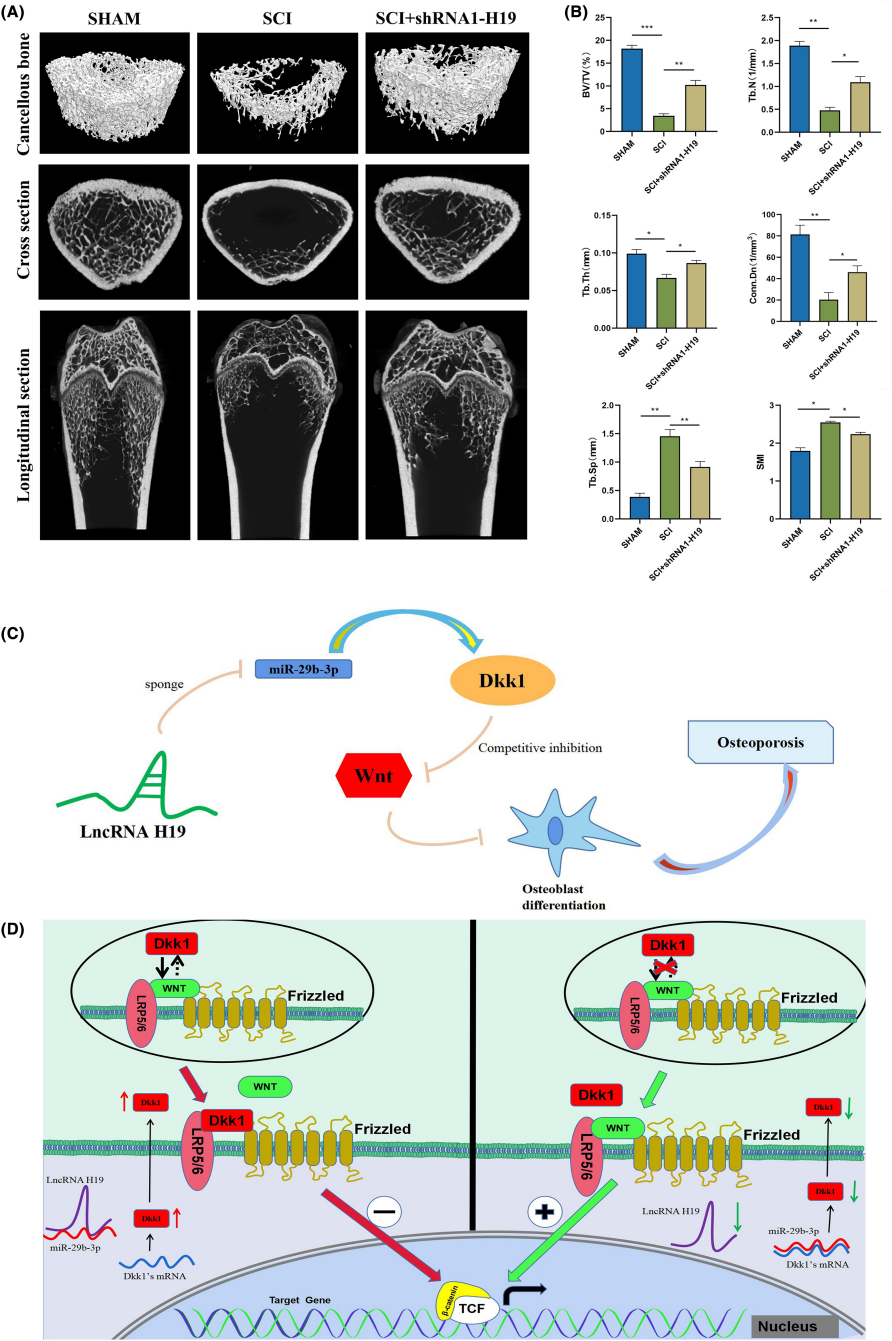
BMSCs transfected with shRNA1‐H19 effectively delay the progression of osteoporosis after spinal cord injury. (A) Schematic diagram of micro‐CT analysis of distal femur (working voltage = 70 kV, working current = 57 μA, scanning resolution = 7 μm). (B) Quantitative analysis of bone parameters (BV/TV and Tb. N, Tb. Th, Conn. Dn, SMI, Tb. SP). (C) Schematic diagram of the H19‐miR‐29b‐3p‐DKK1 axis regulating BMSCs mediated osteoporosis after spinal cord injury. Schematic diagram of the lncRNA H19‐miR‐29b‐3p‐DKK1 axis that regulates osteogenic differentiation of BMSCs. (D) lncRNA H19 sponge miR‐29b‐3p reduces the targeting effect of miR‐29b‐3p and DKK1's mRNA, leading to an increase in the expression of DKK1, further competing with Wnt, thereby inhibiting the Wnt/β‐catenin signal pathway. However, this inhibitory effect was alleviated when the H19 expression was reduced. Data are presented as the means ± SEM (*n* = 6 per group). *p < 0.05, **p < 0.01, ***p ＜ 0.001; VS SCI group.

We generated osteoporotic rats with SCI and administered them with BMSCs transfected with shRNA1‐H19 to investigate whether modulating the level of lncRNA H19 could enhance the therapeutic potential of BMSCs for clinical application. Micro‐CT scanning revealed cortical bone thickness increase and an increase in bone trabecularity in the treatment group compared with that in the SCI group (Figure 8A). The quantitative study showed that the SMI and Tb.Sp values decreased but did not drop to the levels observed in the SHAM group, whereas the BV/TV, Tb.N, Tb.Th and Conn.Dn values increased compared to those in the SCI group (Figure 8B). Based on these findings, we deduced that transplantation of BMSCs transfected with shRNA1‐H19 can prevent osteoporosis from progressing after spinal cord damage.

## 4 DISCUSSION

Osteoporosis is a common bone disease that causes thousands of fractures every year.^27^, ^28^ An imbalance between the new bone and bone resorption capacities is the main cause of osteoporosis.^20^ The complex and carefully coordinated interactions between transcriptional networks and the regulation of signalling pathways in the intra‐tissue environment make it difficult to develop treatment options for osteoporosis.^20^ It is now known that multiple factors can lead to osteoporosis, and in different types of osteoporosis, there may be slight differences in the molecular mechanisms of their pathological progression. In this study, we focused on the rapidly progressive and severe types of osteoporosis after SCI. To investigate this, a combination of in vitro and in vivo techniques was used.

Recently, it has been widely acknowledged that lncRNAs are essential for controlling pathological progression associated with osteoporosis.^26^ The role of H19 in osteoporosis has been extensively studied. H19 is reduced in BMSCs of ovariectomized mice and is associated with Foxc2 synergistically promoted osteogenic differentiation of BMSCs via Wnt/β‐catenin pathway.^29^ Knockout of H19 impairs the proliferation and osteogenic differentiation of BMSCs, while enhancing lipid droplet formation in preadipocytes.^30^ Currently, research on osteoporosis after SCI is still limited.^23^, ^31^ Consistent with the results of previous studies, we found that H19 expression was significantly upregulated in the SCI group.^24^ BMSCs isolated from rats with SCI show lower activity and osteogenic ability.^32^ However, H19 knockout significantly improved the ability of BMSCs to differentiate into osteoblasts, contradicting the findings of previous studies.^23^, ^31^ The different types of osteoporosis, changes in levels of certain factors within the organism and differences in downstream target genes may explain this phenomenon. However, further research is required to confirm these findings.

Numerous studies have shown that miRNAs influence important osteogenic differentiation factors in BMSCs, thereby regulating the biological processes involved in osteogenic differentiation.^25^, ^26^ In a population with osteoporosis, miR‐29b‐3p expression was dramatically downregulated according to a bioinformatics study (https://ncbi.nlm.nih.gov/geo/geo2r/?acc=GSE74209). However, there is currently no evidence that miR‐29b‐3p mediates the development of osteoporosis after SCI. Therefore, in this study, we identified for the first time that miR‐29b‐3p expression is significantly reduced in BMSCs with osteoporosis after SCI. BMSCs treated with the miR‐29b‐3p mimic showed enhanced osteogenic differentiation ability, while knocking out miR‐29b‐3p showed the opposite result.

In osteoporosis studies, there is a close relationship between the regulatory axis composed of lncRNA and miRNA and the Wnt/β‐catenin signalling pathway.^33^, ^34^, ^35^ Studies in the past suggested that inhibiting the Wnt/β‐catenin signalling pathway might reduce osteogenic development.^36^ Given that their increased expression greatly diminishes the Wnt/β‐catenin signalling pathway's favourable effects on osteogenic growth, GSK‐3β and DKK1 are regarded as negative regulatory factors in this pathway.^37^, ^38^ In this experiment, it was found that the increase in miR‐29b‐3p reduced the expression of GSK‐3β and DKK1. The target binding sites between miR‐29b‐3p and DKK1 were determined through bioinformatics analysis, indicating the possible existence of targeted binding interactions between these two molecules. However, no binding sites were found between miR‐29b‐3p and GSK‐3β (https://mirdb.org/cgi‐bin/search.cgi). Meanwhile, through bioinformatics analysis, miR‐29b‐3p was predicted as a downstream target gene for H19 (https://bibiserv.cebitec.uni‐bielefeld.de/rnahybrid?id=rnahybrid_view_submission). Two dual‐luciferase reporter gene experiments confirmed the targeted binding of miR‐29b‐3p to H19 and DKK1. In addition, rescue experiments showed that low miR‐29b‐3p expression eliminated the impact of H19 low expression on osteogenic differentiation. Therefore, we propose that H19 controls the expression of DKK1 by targeting miR‐29b‐3p, thereby controlling the osteogenic differentiation of BMSCs. In addition, we conducted an in vivo experiment that is widely used because of the ability of BMSCs to repair damaged tissues.^12^ We injected shRNA‐H19 lentivirus‐transfected BMSCs into rats with osteoporosis after SCI. Compared with the untreated group, the treatment group showed better relief from bone mineral loss.

We investigated the regulatory functions and mechanisms of miR‐29b‐3p and H19 in controlling osteogenic development of BMSCs during osteoporosis following SCI. Thus, miR‐29b‐3p and H19 may serve as reliable indicators of osteoporosis. However, the specific molecular processes underlying miR‐29b‐3p and H19 and their upstream regulatory networks remain unknown. Our study did not involve intervention validation in transgenic animals, which is a limitation. The research design and methods used in this study are similar to those used by Li et al.^25^ However, in‐depth in vivo animal research is crucial for interpreting experimental results. We established that H19 acts as a sponge to prevent miR‐29b‐3p from attaching to DKK1 mRNA. Overexpression of miR‐29b‐3p or silencing of H19 may be effective strategies to promote bone regeneration. Further studies are necessary to completely understand the physiological mechanisms that cause H19 and miR‐29b‐3p to be released into the circulation through bone tissue in patients. Our results highlight the importance of the regulatory function of the H19‐miR‐29b‐3p‐DKK1 axis in regulating BMSCs' capacity for osteogenic differentiation via the Wnt/β‐catenin signalling pathway (Figure 8C,D). These findings may provide a better understanding and treatment for osteoporosis after SCI.

In conclusion, the pathophysiology of osteoporosis following SCI is significantly influenced by the H19/miR‐29b‐3p/DKK1 axis. This axis affects the Wnt/β‐catenin signalling system, which in turn affects the ability of BMSCs to differentiate into osteoblasts.

## AUTHOR CONTRIBUTIONS

**Sen Qin:** Writing – original draft (lead); writing – review and editing (lead). **Da Liu:** Funding acquisition (equal).

## CONFLICT OF INTEREST STATEMENT

Sen Qin and Da Liu announced that they have no conflicts of interest.

## Supporting information


Table S1.

Table S2.

## Data Availability

The datasets used and/or analysed in the current study are available from the corresponding author upon reasonable request.
