# Supplementary material for: Long non‐coding RNA H19 mediates osteogenic differentiation of bone marrow mesenchymal stem cells through the miR‐29b‐3p/DKK1 axis
Source: J Cell Mol Med. 2024 Apr 29;28(9):e18287. doi: 10.1111/jcmm.18287 (PMC11058329; doi:10.1111/jcmm.18287)
Supplement: Supplementary file 1 — Table S1. [file JCMM-28-e18287-s002.docx]

**Table S1**  Primary antibodies used for the detection of protein expression

| Name | Manufacturer | Dilution ratio:  (Western blot; Immunofluorescence) |
| --- | --- | --- |
| Runx2 | Affinity, Liyang, China  (AF5186) | 1:2000; 1:300 |
| OSX | Affinity, Liyang, China  (DF7713) | 1:3000 |
| OPN | Affinity, Liyang, China  (AF0227) | 1:3000 |
| OCN | Affinity, Liyang, China  (DF12303) | 1:2000 |
| β-catenin | Affinity, Liyang, China  (AF6266) | 1:2000 |
| TCF-1 | Proteintech,Wuhan,China  (22426-1-AP) | 1:2000 |
| GSK-3β | Affinity, Liyang, China  (AF5016) | 1:2000 |
| Dkk1 | Affinity, Liyang, China  (AF4600) | 1:2000; 1:200 |
| Goat Anti-Rabbit IgG | Abbkine, Wuhan, China  (A21020) | 1:30000 |
| GAPDH | Affinity, Liyang, China  (AF7021) | 1:10000 |
