# Supplementary material for: Long non‐coding RNA H19 mediates osteogenic differentiation of bone marrow mesenchymal stem cells through the miR‐29b‐3p/DKK1 axis
Source: J Cell Mol Med. 2024 Apr 29;28(9):e18287. doi: 10.1111/jcmm.18287 (PMC11058329; doi:10.1111/jcmm.18287)
Supplement: Supplementary file 2 — Table S2. [file JCMM-28-e18287-s001.docx]

**Table S2** **The siRNA, inhibitor and mimics sequences**

| **Name** | **Sequences** |
| --- | --- |
| **si-H19-01** | Sense: 5′-GGACAGUUAGCAAAGGAGAdTdT-3′  Antisense: 5’-UCUCCUUUGCUAACUGUCCdTdT-3’ |
| **si-H19-02** | Sense: 5′-GGUUGAAGGGCCUGAGCUAdTdT-3′  Antisense: 5’-UAGCUCAGGCCCUUCAACCdTdT-3’ |
| **si-H19-03** | Sense: 5′-GGAUGACAGGUGUGGUCAAdTdT-3′  Antisense: 5’-UUGACCACACCUGUCAUCCdTdT-3’ |
| **si-NC** | Sense: 5’-UUCUCCGAACGUGUCACGUTT-3’  Antisense: 5’- ACGUGACACGUUCGGAGAATT-3’ |
| **rno-miR-29b-3p mimics** | Sense: 5′-UAGCACCAUUUGAAAUCAGUGUU-3′  Antisense: 5’-AACACUGAUUUCAAAUGGUGCUA-3’ |
| **Mimics-NC** | Sense: 5’-UUCUCCGAACGUGUCACGUTT-3’  Antisense: 5’- ACGUGACACGUUCGGAGAATT-3’ |
| **rno-miR-29b-3p inhibitor** | 5′-AACACUGAUUUCAAAUGGUGCUA-3′ |
| **inhibitor-NC** | 5′-CAGUACUUUUGUGUAGUACAA-3′ |
